# Severe skull base osteomyelitis caused by *Pseudomonas aeruginosa* with successful outcome after prolonged outpatient therapy with continuous infusion of ceftazidime and oral ciprofloxacin: a case report

**DOI:** 10.1186/s13256-017-1221-7

**Published:** 2017-02-21

**Authors:** Cristina Conde-Díaz, Jara Llenas-García, Mónica Parra Grande, Gertrudis Terol Esclapez, Mar Masiá, Félix Gutiérrez

**Affiliations:** 1 Infectious Diseases Unit, Hospital General Universitario de Elche, Camino de la Almazara, 11, 03203 Elche, Alicante Spain; 2 Foundation for the Promotion of Health and Biomedical Research in the Valencian Region (FISABIO), Valencia, Spain; 3 Departement of Microbiology, Hospital General Universitario de Elche, Alicante, Spain; 4 Universidad Miguel Hernández, Alicante, Spain

**Keywords:** Skull base osteomyelitis, Cranial nerve palsy, *Pseudomonas aeruginosa*, Malignant external otitis, Outpatient parenteral antibiotic therapy

## Abstract

**Background:**

Skull base osteomyelitis is an uncommon disease that usually complicates a malignant external otitis with temporal bone involvement. It affects predominantly diabetic and immunocompromised males and has a high mortality rate. *Pseudomonas aeruginosa* is the most common causative organism. Currently, there is no consensus about the best therapeutic option. Here we describe a case of severe skull base osteomyelitis caused by *Pseudomonas aeruginosa* with progressive palsy of cranial nerves that was successfully managed with prolonged outpatient continuous infusion of ceftazidime plus oral ciprofloxacin.

**Case presentation:**

A 69-year-old Caucasian man presented with dysphagia, headache, and weight loss. He complained of left earache and purulent otorrhea. Over the following weeks he developed progressive palsy of IX, X, VI, and XII cranial nerves and papilledema. A petrous bone computed tomography scan showed a mass in the left jugular foramen with a strong lytic component that expanded to the cavum. A biopsy was then performed and microbiological cultures grew *Pseudomonas aeruginosa*. After 6 weeks of parenteral antibiotic treatment, our patient was discharged and treatment was continued with a domiciliary continuous infusion of a beta-lactam through a peripherally inserted central catheter, along with an oral fluoroquinolone for 10 months. Both radiological and clinical responses were excellent.

**Conclusions:**

Skull base osteomyelitis is a life-threating condition; clinical suspicion and correct microbiological identification are key to achieve an accurate and timely diagnosis. Due to the poor outcome of *Pseudomonas aeruginosa* skull base osteomyelitis, prolonged outpatient parenteral antibiotic therapy administered by continuous infusion could be a valuable option for these patients.

## Background

Skull base osteomyelitis (SBO) is a rare disease with a high mortality rate, up to 10–20% [1]. It was first described in 1959 by Meltzer and Keleman [2]. It affects predominantly diabetic or immunocompromised men in their sixties. Typical cases arise as a complication of malignant external otitis with temporal bone involvement. *Pseudomonas aeruginosa* is the causative organism in 75–95 % of cases [3]. However, there are also atypical cases of central SBO that usually affect sphenoid and/or occipital bones and that are not associated with malignant external otitis. Main clinical features are otalgia, facial pain, and headache (90%). Facial nerve palsy complicates around 60% of cases [4]. Venous sinus thrombosis, meningitis, cerebritis or abscess formation are less frequent complications [5]. Early treatment is key to success. Empiric treatment should be based on anti-*Pseudomonal* agents [6]. Optimal treatment is not clear; usually a course of intravenous treatment is followed by a course of oral antibiotics with a highly variable duration [7]. Here we describe a case of a patient with SBO caused by *Pseudomonas aeruginosa* with extensive soft tissue and bone involvement complicated with progressive palsy of cranial nerves, which was initially misdiagnosed as malignancy and was managed with prolonged domiciliary parenteral therapy.

## Case presentation

A 69-year-old Caucasian man was admitted to hospital with a 2-week history of dysphagia to both liquids and solids, headache, and weight loss. His medical history was remarkable for hypertension and type 2 diabetes mellitus treated with oral antidiabetics. Three months prior to the hospital admission, he complained of otalgia and left ear discharge; therefore, a diagnosis of otitis externa was made and he was given ear drops containing antibiotics.

**Fig. 1:**
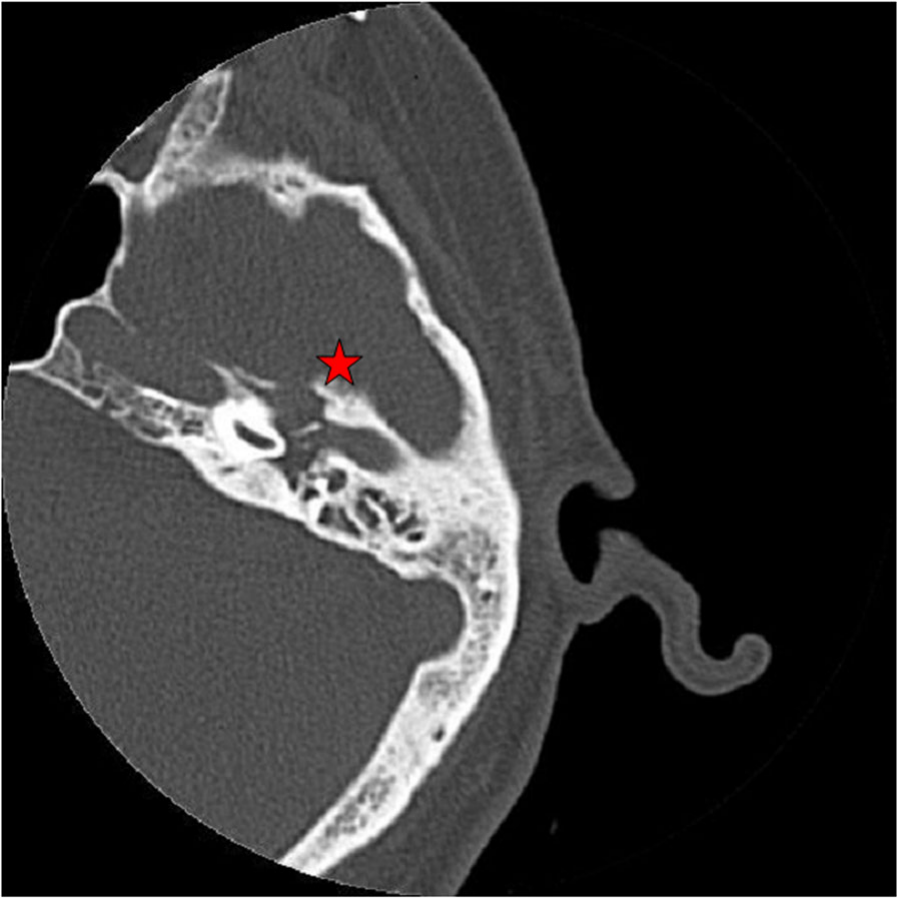
Petrous bone computed tomography scan shows occupation of the left middle ear and mastoid cells and erosion of the anterior wall of the middle ear (*star*)

On physical examination his vital signs were normal and he had no fever. A neurological examination revealed absence of nausea reflex. There was no evidence of other cranial nerve palsies and a peripheral neurological examination was normal. Purulent otorrhea and a bulging left tympanic membrane were seen through the otoscope. The examination was otherwise unremarkable. Initial blood test results showed mild hyponatremia (132 mEq/L) and an elevated C-reactive protein (CRP) (88 mg/L) and erythrocyte sedimentation rate (ESR) (104 mm/h). A petrous bone computed tomography (CT) scan and a cranial magnetic resonance imaging (MRI) scan showed a vascularized mass in the left jugular foramen, extending to the middle ear and breaking its front wall (Fig. 1). The findings were suggestive of a jugulo-tympanic glomus, but it was finally ruled out because of a normal arteriography. An esophagogastroduodenoscopy was also normal. Since the main suspicion was a primary or metastatic cancer, a tympanostomy was performed and a middle ear biopsy result was negative for malignancy.

**Fig. 2:**
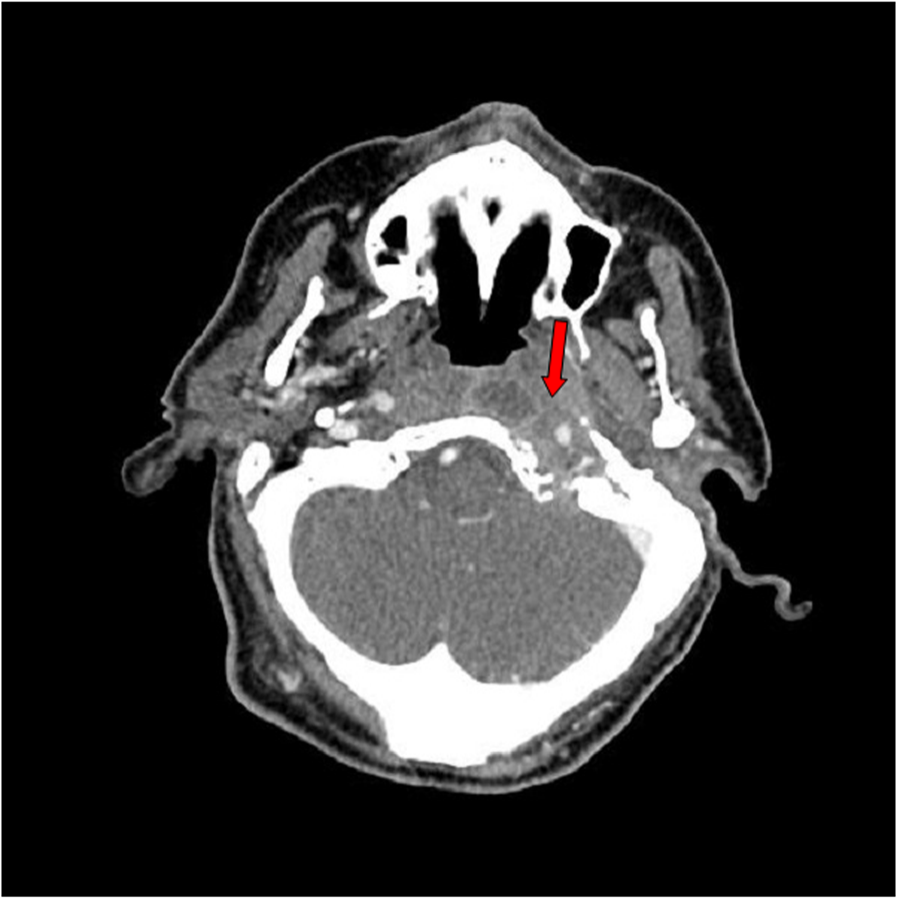
Cranial computed tomography scan shows a mass of soft tissue (*arrow*) surrounding the internal jugular vein and the carotid artery at the left jugular foramen with signs of bone erosion and destruction. The lesion extends medially and causes bone destruction of the left occipital condyle and the left side edge of the clivus and erosion of the posterior edge of the oval hole. In the petrous bone it extends to the middle ear and causes erosion of the anterior wall of the tympanic cavity. The mass goes along the petrous carotid reducing its caliber and causing bone destruction of the anterior edge of the carotid canal extending to the petrous apex

**Fig. 3:**
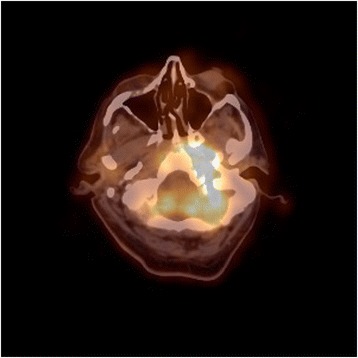
Positron emission tomography scan showing an uptake with a standardized uptake value of 7.4 at the mass location

Two weeks later, our patient was referred for transient visual obscurations, double vision, worsening headache, and vomiting. On neurological examination, binocular diplopia and left hypoglossal nerve palsy were observed, together with ptosis of the left soft palate and paralysis of left arytenoid, suggesting VI, IX, and X cranial nerve palsies. Funduscopy revealed papilledema. A new cranial CT scan showed progression of the left jugular foramen lytic lesion now expanding to the cavum (Fig. 2). A positron emission tomography (PET) scan showed a mass with a standardized uptake value (SUV) of 7.4 (Fig. 3). Intracranial hypertension was suspected, and therefore a diagnostic and therapeutic lumbar puncture performed. Cerebrospinal fluid (CSF) showed 60 leukocytes/μL (81% mononuclear cells), glucose 66 mg/dL, and proteins 189 mg/dL. CSF cultures and nucleic acid testing results were negative, as was CSF cytology. An endoscopic biopsy of the cavum mass was then performed obtaining purulent material. The anatomopathological study showed acute and chronic inflammation and was negative for malignancy; microbiological cultures grew *Pseudomonas aeruginosa*. A final diagnosis of SBO with multiple cranial nerve palsies, intracranial hypertension, aseptic meningitis, and a secondary syndrome of inappropriate antidiuretic hormone secretion (SIADH) was made. Intravenous meropenem (minimal inhibitory concentration [MIC]: ≤1 μg/mL) and ciprofloxacin (MIC: ≤0.5 μg/mL) were initiated. Neurosurgeons did not recommend surgical debridement. After 6 weeks, our patient was discharged with oral ciprofloxacin (750 mg twice daily) along with intravenous ceftazidime (4 g daily; MIC: ≤ 1 μg/mL) given domiciliary as continuous infusion through a peripherally inserted central catheter (PICC). Clinical progress was slow but favorable, with normalization of the acute-phase reactants and no metabolic activity on the PET scan, so antibiotics were stopped after 11 months of parenteral treatment. Despite the prolonged antibiotic therapy, no remarkable side effects were observed. One month after stopping antibiotics, a new PET scan did not show any metabolic activity, and levels of CRP and ESR remained low. All neurological deficits resolved, except for a mild persistent left sixth cranial nerve palsy.

## Discussion

We describe the case of a patient with SBO with multiple and progressive cranial nerve palsies initially misdiagnosed as a malignant lesion. Information about this entity is circumscribed to case reports and small case series [3, 4, 6, 8–11], a systematic review comprising 42 central SBO cases [1], and a meta-analysis of 83 SBO cases [4]. Large tertiary hospitals report around three cases a year [3, 4, 11].

There is some confusion on the terminology and some authors refer to SBO as the entity resulting from complication of a malignant external otitis [6, 8, 9, 12], while others include also the so-called atypical or central SBO. The latter usually affects sphenoid and/or occipital bones and it may be secondary to an otogenic, sinusal, odontogenic or hematogenous infection, although there are also cases with no obvious infective source [3, 4, 7, 10]. Some authors prefer to avoid the term SBO and define instead a lateral temporal bone and a medial temporal bone osteomyelitis [11]. In our case, the SBO was preceded by a malignant external otitis so the infection most probably traversed the fissures of Santorini and the tympanomastoid suture to the compact bone, finally affecting the skull base. In a recent meta-analysis, 59% of patients had some cranial nerve palsy (that persisted after therapy in 41%) while disease-specific mortality reached 22% [4]. The VII cranial nerve is the cranial nerve most commonly affected. Contrary to this, in our patient the VII cranial nerve was not involved and the infection expanded directly inferomedially to the jugular foramen, giving rise to Vernet’s syndrome (glossopharyngeal nerve, vagus nerve and, accessory nerve). As the infection progressed, further involvement of the hypoglossal nerve at its exit at the hypoglossal canal (Collet-Sicard syndrome) was noticed. Involvement of the VI cranial nerve probably occurred at the Dorello’s canal where it passes over the edge of the petrous pyramid.

*Pseudomonas aeruginosa* is the principal causative microorganism of SBO, although other bacteria like *Staphylococcus aureus*, *Klebsiella* spp., *Proteus* spp., and *Mycobacterium chelonae* have been reported [1, 4]. Fungal infection also needs to be considered, especially in patients with underlying chronic sinusitis, sinusal pain, facial/periorbital swelling or in the absence of ear discharge [3]. *Candida*, *Aspergillus*, *Rhizopus*, and *Mucor* have been involved [4]. In the review by Blyth *et al*., *Zygomycetes* were responsible for more than 50% of fungal SBO, and as a result amphotericin B is suggested as the preferred empirical treatment when fungal SBO is suspected [3].

CRP and ESR are often used as a diagnostic adjunct in SBO and especially for the follow-up of patients. Imaging examinations such as CT and MRI are crucial to characterize the lesion and to evaluate the degree of bone and soft tissue involvement. The differential diagnosis of soft tissue masses at the skull base is wide and comprise malignant lesions (nasopharyngeal carcinoma, squamous cell carcinoma of the external auditory canal, schwannoma, metastases, and multiple myeloma), infectious and inflammatory diseases (SBO, inflammatory pseudotumor, granulomatosis with polyangeitis, tuberculosis, and sarcoidosis) and others, such as cholesteatoma, dural arteriovenous fistula, fibrous dysplasia, and Paget disease [1, 5, 10, 13]. The use of imaging tests that measure the activity of infection also has a role in the diagnosis and follow-up of these patients. The use of single-photon emission tomography-computed tomography (SPECT-CT) with ^67^Ga or ^111^In-labeled leukocytes has shown higher specificity than scintigraphy, CT scan or SPECT alone [14]. In a recent study by Sharma *et al*., SPECT-CT was the most sensitive technique for SBO diagnosis (sensitivity = 100%, specificity = 50%), while CT was the most specific (sensitivity = 73%, specificity = 100%) [15]. In a study by Filippi *et al*., specificity of SPECT-CT was higher than SPECT alone (89% vs. 78%) [16]. SPECT scan has also shown good predictive value for long-term outcomes [9]. Obtaining tissue specimens is paramount to determine the microbial etiology and needs proper microbiological and histopathological processing of samples. In our case, improper management of initial middle ear samples (that were not sent to the microbiology laboratory) may have contributed to the diagnostic delay.

Empiric treatment of SBO should include broad-spectrum antibiotics with good anti-*Pseudomonas* activity, evaluating the need of added antifungal therapy [6]. Treatment duration and route of administration are not well-resolved issues and a wide variety of therapeutic approaches have been adopted in previously reported cases. Initial intravenous treatment is usually followed by long-term oral therapy, providing an adequate clinical response is achieved with intravenous antibiotics and active oral agents exist. However, *Pseudomonas aeruginosa* osteomyelitis has been associated with lower cure rates and higher recurrence risk [17], therefore antimicrobial therapy has been continued domiciliary for several weeks to minimize relapses [7, 18]. In the meta-analysis by Ridder *et al*., median length of inpatient treatment was 32 days (range: 7–83), while median length of subsequent oral treatment was 1.5 months (range 0–16 months) [4]. Antibiotic regimens comprising an initial 4–6 weeks course of intravenous treatment followed by 6 to 12 months of oral medication, guided by clinical response, have been recommended [7]. Continuous infusion of antibiotics allows outpatient administration of parenteral therapy, thereby reducing health care resources consumption [19]. Administration of beta-lactams by continuous intravenous infusion generates higher blood and tissue concentrations and allows longer time above the MIC compared with intermittent dosing, which is particularly useful for bacteria with high MIC values, such as *Pseudomonas aeruginosa* [20]. Because of the wide soft tissue and bone involvement, the critical location of the abscesses, the lack of adjunct surgical debridement, the Pseudomonal etiology, and the absence of side effects, we decided to give the patient a prolonged course of intravenous ceftazidime with domiciliary continuous infusion through a PICC, along with oral ciprofloxacin. Our strategy was successful, allowing for prolonged outpatient intravenous treatment. Remarkably, no side effects were noticed after 11 months of beta-lactam and fluoroquinolone treatment. We believe this is a valid approach, which may be crucial in cases of drug-resistant microorganisms with limited therapeutic options.

The role of hyperbaric oxygen is not well defined, but may provide some benefit reversing the hypoxia, improving the phagocytic activity against aerobic microorganisms, and stimulating angiogenesis; a positive effect on the cranial nerve palsies recovery has been reported [4, 21, 22]. Surgery may be needed for decompressing cranial nerves in selected cases [11]. The role of surgical debridement remains controversial. It has no clear impact on survival, antimicrobial treatment duration nor prognosis [9, 18, 21], except in the case of fungal SBO [3]. Nevertheless, surgical debridement could remove devitalized tissue and reduce infection load in order to improve penetration of antibiotics and some authors advocate for an early and forceful surgical approach, especially in patients with protracted ear infections and signs of cranial nerve involvement [4].

Close radiological and clinical follow-up of SBO is mandatory. Follow-up gallium scans have been proposed at 1 week and 12 weeks after antibiotics cessation to search for recurrence [22].

## Conclusions

SBO can be misdiagnosed as malignancy, especially when there is a strong lytic bone destruction and important soft tissue involvement, as in the reported case. Clinical suspicion, even in the absence of an obvious infective source, and microbiological processing of samples are key to achieve a correct and timely diagnosis. *Pseudomonas aeruginosa* is the most frequent causative organism. Outpatient parenteral antibiotic therapy administered by continuous infusion could be a suitable option to prolong intravenous treatment in this severe disease, especially when difficult-to-treat organisms like *Pseudomonas aeruginosa* are implicated.

## Abbreviations

CRP: C-reactive protein
CSF: Cerebrospinal fluid
CT: Computed tomography
ESR: Erythrocyte sedimentation rate
MIC: Minimal inhibitory concentration
MRI: Magnetic resonance imaging
PET: Positron emission tomography
PICC: Peripherally inserted central catheter
SBO: Skull base osteomyelitis
SIADH: Syndrome of inappropriate antidiuretic hormone secretion
SPECT-CT: Single-photon emission tomography-computed tomography
